# Lipid Droplet–Mitochondria Contacts in Health and Disease

**DOI:** 10.3390/ijms25136878

**Published:** 2024-06-22

**Authors:** Hongjun Fan, Yanjie Tan

**Affiliations:** Center for Cell Structure and Function, Shandong Provincial Key Laboratory of Animal Resistance Biology, Collaborative Innovation Center of Cell Biology in Universities of Shandong, College of Life Sciences, Shandong Normal University, Jinan 250014, China; fanhongjun0913@163.com

**Keywords:** lipid droplet, mitochondria, metabolism, redox, disease

## Abstract

The orchestration of cellular metabolism and redox balance is a complex, multifaceted process crucial for maintaining cellular homeostasis. Lipid droplets (LDs), once considered inert storage depots for neutral lipids, are now recognized as dynamic organelles critical in lipid metabolism and energy regulation. Mitochondria, the powerhouses of the cell, play a central role in energy production, metabolic pathways, and redox signaling. The physical and functional contacts between LDs and mitochondria facilitate a direct transfer of lipids, primarily fatty acids, which are crucial for mitochondrial β-oxidation, thus influencing energy homeostasis and cellular health. This review highlights recent advances in understanding the mechanisms governing LD–mitochondria interactions and their regulation, drawing attention to proteins and pathways that mediate these contacts. We discuss the physiological relevance of these interactions, emphasizing their role in maintaining energy and redox balance within cells, and how these processes are critical in response to metabolic demands and stress conditions. Furthermore, we explore the pathological implications of dysregulated LD–mitochondria interactions, particularly in the context of metabolic diseases such as obesity, diabetes, and non-alcoholic fatty liver disease, and their potential links to cardiovascular and neurodegenerative diseases. Conclusively, this review provides a comprehensive overview of the current understanding of LD–mitochondria interactions, underscoring their significance in cellular metabolism and suggesting future research directions that could unveil novel therapeutic targets for metabolic and degenerative diseases.

## 1. Introduction

The intricate orchestration of cellular metabolism and redox balance is fundamental to the sustenance of life. At the cellular level, this balance is maintained by a complex network of biochemical pathways and interactions between various organelles. Among these, lipid droplets (LDs) and mitochondria have emerged as key players, not just in their traditionally recognized roles but also in their dynamic interactions, which are pivotal for cellular health and function [1,2].

Historically perceived as mere fat storage bodies, LDs have been redefined over the past decade. They are now recognized as highly dynamic organelles with crucial roles in cellular lipid metabolism, signaling, and homeostasis [3,4]. Composed of a core of neutral lipids, primarily triglycerides and esterified cholesterol, surrounded by a phospholipid monolayer and specific proteins, LDs vary in size, number, and distribution depending on the cell type and metabolic state [5]. The biogenesis of LDs is a complex process, starting from the endoplasmic reticulum (ER), where lipid esterification occurs, followed by the budding of these lipid-enriched domains [6]. Mitochondria, often termed the powerhouses of the cell, are central to energy production through oxidative phosphorylation and the tricarboxylic acid cycle [7]. Beyond energy production, they are involved in various metabolic pathways, including fatty acid oxidation, amino acid metabolism, and the synthesis of iron–sulfur clusters [8]. Mitochondria are also crucial in regulating apoptosis and cellular redox balance [9]. Mitochondrial dynamics, including fusion, fission, and biogenesis, are tightly regulated processes essential for maintaining mitochondrial function and integrity [10].

The interaction between LDs and mitochondria represents a critical nexus in cellular metabolism. These contacts facilitate the transfer of fatty acids from LDs to mitochondria, where they undergo β-oxidation, thus linking energy storage with energy utilization [11,12]. This interaction is not merely physical but involves a complex network of protein–protein interactions and signaling pathways [13]. Proteins like perilipin, located on the LD surface, and mitochondrial outer membrane proteins play a pivotal role in mediating these contacts [14]. Recent studies using advanced imaging techniques have provided insights into the dynamics of these interactions, revealing that they are highly regulated and responsive to the cellular metabolic status [15]. The physiological relevance of LD–mitochondria contacts extend beyond mere energy metabolism. These interactions are crucial for maintaining cellular energy homeostasis, especially under conditions of fluctuating energy demand or nutrient availability [16]. They also play a significant role in lipid metabolism, including the synthesis and breakdown of fatty acids, phospholipids, and cholesterol [17]. In adipocytes, for instance, these interactions regulate lipolysis and lipid storage, directly impacting systemic energy balance [18]. Moreover, the cross-talk between LDs and mitochondria is vital to maintaining redox balance within cells [19]. Mitochondrial β-oxidation generates reactive oxygen species (ROS), which can be deleterious at high concentrations. LDs can sequester some of these ROS, thereby mitigating oxidative stress [20].

Dysregulation in LD–mitochondria interactions has been implicated in various metabolic diseases. In obesity and diabetes, altered lipid metabolism and insulin resistance are linked to dysfunctional LD–mitochondria cross-talk [21,22]. In non-alcoholic fatty liver disease, excess lipid accumulation in hepatocytes and subsequent metabolic stress are a result of impaired LD–mitochondria interactions [12,14]. Additionally, there is emerging evidence linking these dysregulated interactions to cardiovascular diseases and potentially to neurodegenerative disorders, where altered metabolism and redox balance play a critical role [17,23]. Given their central role in metabolism and disease, targeting LD–mitochondria interactions present a novel therapeutic strategy. Understanding the molecular mechanisms governing these interactions could lead to the development of drugs aimed at restoring or modulating these contacts, thereby offering potential treatments for metabolic diseases and beyond.

## 2. Basics of LDs

**Figure 1:**
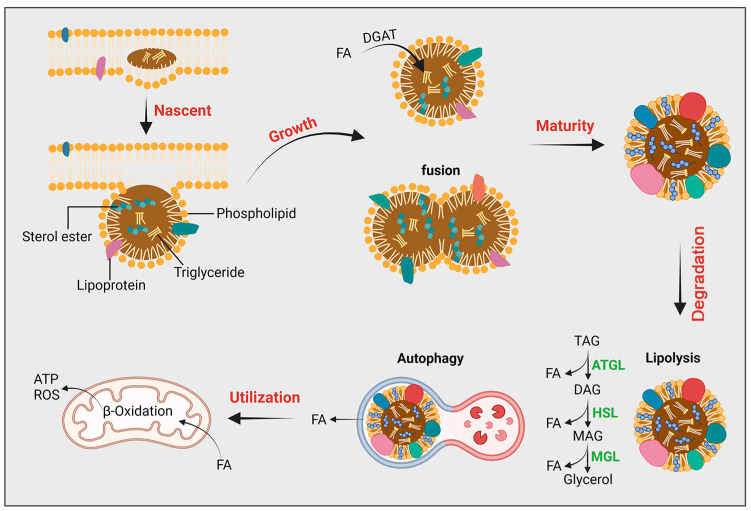
Composition and metabolic process of lipid droplets: LDs are unique cellular organelles composed of a monolayer of phospholipids surrounding a core of neutral lipids (triglycerides, cholesterol, sterols). They originate from the endoplasmic reticulum, initially accumulating as lens-like structures in the ER membrane and ultimately released into the cytoplasm via budding. Free LDs grow through fusion or autonomous growth, leading to the formation of mature LDs. The surface of LDs contains lipolytic enzymes, activated during starvation, which hydrolyze neutral lipids into fatty acids. Additionally, LDs can be targeted and broken down by autophagolysosomes, releasing fatty acids that undergo beta-oxidation in mitochondria to provide energy. TAG: Triacylglycerol; DAG: Diacylglycerol; MAG: Monoacylglycerol; ATGL: Adipose triglyceride lipase; HSL: Hormone-sensitive lipase; MGL: Monoacylglycerol lipase.

LDs, once regarded as mere lipid storage units, have emerged as dynamic organelles pivotal to a myriad of cellular processes [24] (Figure 1). This redefined understanding has expanded our view of LDs from passive lipid reservoirs to active participants in cellular metabolism, signaling, and disease pathogenesis.

### 2.1. LDs’ Structural Characteristics

LDs are characterized by a core of neutral lipids, predominantly triglycerides and esterified cholesterol, encased in a monolayer of phospholipids interspersed with proteins [25]. This unique structure distinguishes LDs from other organelles, which typically possess bilayer membranes. The size and number of LDs vary considerably between cell types and are influenced by metabolic conditions. The protein composition of LDs is diverse and cell-specific. Proteins like perilipins, which coat the LD surface, play crucial roles in regulating lipid metabolism and LD dynamics [26].

### 2.2. LDs’ Formation and Growth

The genesis of LDs is intricately linked to the ER. Lipid esterification, primarily triglyceride synthesis, is catalyzed by enzymes such as diacylglycerol acyltransferase (DGAT) at the ER. This process leads to the accumulation of neutral lipids between the leaflets of the ER membrane, eventually budding off to form nascent LDs [27]. The exact mechanisms governing LD formation and the role of cytosolic factors in this process remain active areas of research.

LD growth can occur through several mechanisms: de novo synthesis of lipids, uptake of external lipids, and fusion of existing LDs. Lipid transport proteins, such as fatty acid-binding proteins, and lipases contribute to these processes, regulating the size and lipid composition of LDs. Maintenance of LD integrity is also crucial. Proteins associated with LDs, including members of the perilipin family, orchestrate these dynamics, mediating interactions with lipases and other regulatory proteins [28].

### 2.3. LDs’ Functional Diversity

LDs are central to energy homeostasis, serving as reservoirs for energy-rich lipids. During periods of energy surplus, triglycerides are stored in LDs; conversely, in times of demand, these triglycerides are mobilized for energy production. LDs also play a significant role in lipid metabolism, participating in the synthesis and degradation of various lipid species. Furthermore, LDs interact with other organelles, including mitochondria, peroxisomes, and lysosomes, facilitating lipid exchange and signaling events [29]. The dynamics of LDs are influenced by a variety of factors. Hormonal cues such as insulin and glucagon modulate LD metabolism in response to the body’s nutritional status. Cellular stress conditions, such as hypoxia and oxidative stress, also impact LD behavior, with LDs serving as buffers against cellular damage. Genetic and epigenetic factors further dictate the formation, size, and number of LDs, highlighting the complexity of LD regulation [30,31,32].

Beyond their metabolic functions, LDs are increasingly recognized as players in intracellular signaling pathways. They influence the activity of transcription factors and gene expression, affecting various cellular processes. LDs also serve as platforms for protein modification and interaction, contributing to the regulation of signaling cascades [33]. LDs are implicated in a range of metabolic disorders. In obesity and diabetes, aberrant LD metabolism and insulin resistance are closely linked. LDs also play a role in the pathogenesis of liver diseases, including nonalcoholic fatty liver disease (NAFLD) and steatohepatitis. Additionally, emerging research points to the involvement of LDs in immune responses, cancer metabolism, and neurodegenerative diseases, underscoring their wide-ranging impact on health and disease [34,35,36].

## 3. Interplay between LDs and Mitochondria

The cellular interplay between LDs and mitochondria represents a critical nexus in the regulation of energy homeostasis and metabolic health. This dynamic interaction is fundamental to mediating lipid metabolism, signaling pathways, and energy balance within cells [11,37].

### 3.1. Peridroplet Mitochondria

Numerous investigations have noted both stable and transient interactions between LDs and mitochondria [16,38,39,40,41,42,43]. The type of interaction—whether stable or dynamic—can vary depending on the specific tissue or organ. For instance, in tissues with high metabolic activity, such as the liver and muscles, dynamic interactions may be more prevalent to rapidly respond to metabolic demands [1,44]. Conversely, in adipose tissue, stable interactions might be more prominent to facilitate sustained lipid storage and regulation [11,39]. This tissue-specific variability underscores the complexity and specialization of metabolic processes in different physiological contexts. Notably, recent findings have highlighted that mitochondria associated with LDs, known as peridroplet mitochondria (PDM), display unique bioenergetic, proteomic, cristae structure, and dynamic properties distinct from cytoplasmic mitochondria [45]. These PDM were isolated from mature brown adipose tissue (BAT) using high-speed centrifugation to separate the mitochondria from their associated LDs. Research by Benador et al. revealed that PDM possess a higher capacity for oxidizing pyruvate and malate, yet a lower capacity for lipid oxidation compared to cytoplasmic mitochondria. Additionally, PDM demonstrate increased ATP synthesis capacity and elevated ATP synthase expression levels. This ATP production in PDM aids in lipid esterification and LD expansion [39]. Moreover, despite high levels of the mitochondrial fusion protein Mfn2, PDM exhibit different dynamics from cytoplasmic mitochondria, as they do not fuse or share contents with neighboring mitochondria [12]. Their reduced motility, likely due to their attachment to LDs, may explain their decreased fusion activity. Data from experiments using the PDM tethered chain protein overexpression model in native brown adipocytes suggest that anchoring mitochondria to LDs is sufficient to impart the distinctive bioenergetic traits observed in PDM [46].

Brown fat plays an important role in thermogenesis, and the interaction of LDs with mitochondria in brown fat has a potential effect on thermogenesis. A previous study showed that in brown adipose tissue, exposure to cold environments leads to simultaneous increases in the expressions of UCP1 and PLIN5, the latter being a protein critical for LD–mitochondria interactions. It has been observed that the upregulation of PLIN5 is dependent on UCP1 expression [47]. Given that PLIN5 is essential for LD–mitochondria interactions, its increased expression under cold conditions enhances these interactions, facilitating the provision of energy. While it is not yet clearly established that LD–mitochondria interactions directly promote thermogenesis, these interactions can potentially expedite lipid transfer and provide the fatty acids necessary for heat production. Therefore, LD–mitochondria interactions may play a role in promoting thermogenesis, though this requires further investigation.

### 3.2. Mechanisms of LD–Mitochondria Contacts

**Figure 2:**
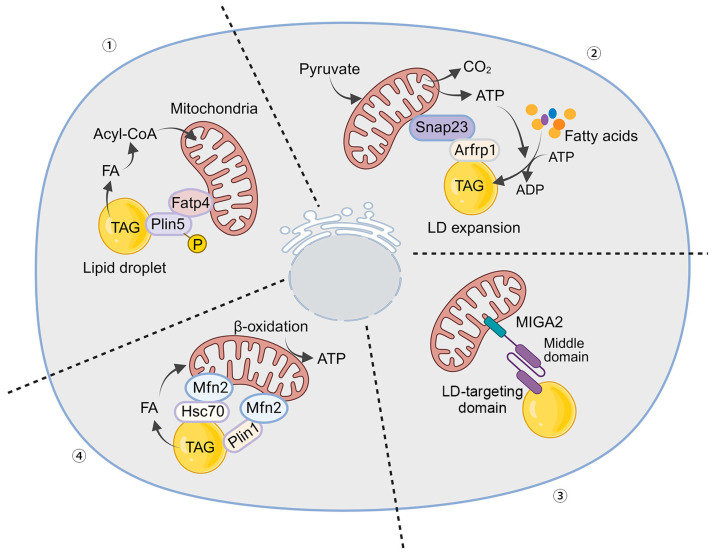
Mechanisms of Mitochondria–LD Interaction. (1) PLIN5 and FATP4 interaction: The C-terminal structural domain of PLIN5 interacts with FATP4, enhancing the connections between LDs and mitochondria. Starvation triggers the phosphorylation of PLIN5, leading to lipolysis and the release of fatty acids from LDs into mitochondria. These fatty acids are then converted to fatty acyl-CoAs for oxidation. (2) ARFRP1 and SNAP23 recruitment: ARFRP1 recruits SNAP23 to a site near the LD, promoting LD–mitochondria interactions and facilitating LD amplification. (3) MIGA2 linkage: The mitochondrial outer membrane protein MIGA2 links mitochondria to LD proteins, enabling efficient lipid storage within the LD. (4) Mfn2 and Hsc70/PLIN1 complex formation: Mitochondria-localized Mfn2 and LD-localized Hsc70 or PLIN1 form a complex at the mitochondria–LD membrane contact site. This complex tethers mitochondria to the LD, facilitating the transfer of fatty acids from LDs to mitochondria for β-oxidation.

The physical interactions between lipid droplets (LDs) and mitochondria are crucial for direct lipid transfer and metabolic communication. These interactions are facilitated by proteins that anchor LDs to the mitochondrial membrane, creating a specialized microenvironment for efficient lipid exchange (Figure 2).

Perilipin 1 (PLIN1) and Perilipin 5 (PLIN5): These proteins are essential to mediating LD interactions with mitochondria. PLIN1 primarily controls lipolysis and lipid storage, while PLIN5 directly facilitates LD–mitochondria contacts. PLIN5 anchors LDs to mitochondria, enabling efficient fatty acid transfer for oxidation [48]. During starvation, phosphorylation of PLIN5 in adult myoblasts promotes the transport of LD-associated fatty acids to mitochondria for β-oxidation, requiring an intact mitochondrial tethering structural domain within PLIN5 [49]. The acyl-CoA synthetase fatty acid transport protein 4 (FATP4) serves as a mitochondrial linker for PLIN5 [15]. The C-terminal structural domains of PLIN5 and FATP4 form the minimal protein interaction domain that induces organelle contacts. Starvation triggers PLIN5 phosphorylation, leading to lipolysis and fatty acid transport from LDs to FATP4 on mitochondria, where they are converted to fatty acyl-CoA for subsequent oxidation [15,50].

Mitofusin 2 (MFN2): As part of the outer mitochondrial membrane, MFN2 is critical in forming contacts with LDs. It not only contributes to mitochondrial fusion but also tethers mitochondria to LDs [51]. A complex involving mitochondria-localized Mfn2 and LD-localized Heat shock cognate 71 kDa protein (Hsc70) is formed at the mitochondria–LD membrane contact site, facilitating fatty acid transfer from LDs to mitochondria for β-oxidation. Lipid overload decreases Mfn2 levels, impeding the mitochondria–LD contacts (MLCs) and leading to lipid accumulation. Restoring Mfn2 levels reestablishes MLCs, reducing myocardial lipotoxicity under lipid overload conditions both in vivo and in vitro. Chronic lipid overloads induce Mfn2 degradation via the ubiquitin–proteasome pathway after acetylation at the K243 site, shifting from adaptive lipid utilization to lipotoxicity. This tethering is vital for lipid transfer during high energy demands like fasting or exercise [46,51].

Acyl-CoA Synthetase Long-Chain Family Member 1 (ACSL1): ACSL enzymes regulate lipid metabolism, including fatty acid elongation, oxidative catabolism, phospholipogenesis, and protein acylation [52]. ACSL1 is crucial for fatty acid metabolism, activating fatty acids for β-oxidation in mitochondria and regulating LD formation and lipolysis. Its activity is closely tied to the cell’s metabolic status and influences LD–mitochondria interactions [53,54].

Synaptosome-associated protein 23 (SNAP23): SNAP23, a SNARE protein, is highly expressed in human skeletal muscle [55]. It mediates insulin-stimulated docking and fusion of glucose transporter 4 (GLUT4) with the plasma membrane [55]. Recent studies suggest that SNAP23 facilitates the import of LD-derived fatty acids into neighboring mitochondria for β-oxidation. Research involving skeletal muscle biopsies from lean, healthy men showed co-localization of SNAP23 with mitochondrial and LD markers [56]. Studies also reported higher levels of SNAP23 associated with LDs in the livers of fasted mice, indicating increased LD–mitochondria interactions. SNAP23 may facilitate lipid and protein transfer between LDs and mitochondria, although the exact mechanisms remain under investigation [57]. Knockdown of SNAP23 with siRNA reduces LD–mitochondria interactions and β-oxidation [56]. Additionally, ADP-ribosylation factor related protein 1 (ARFRP1) recruits SNAP23 near LDs, promoting LD growth in hepatitis C virus-infected cells, suggesting SNAP23’s role in LD amplification [58].

VPS13D and MIGA2: Recent research has identified recombinant Vacuolar Protein Sorting 13D (VPS13D) and mitoguardin 2 (MIGA2) as novel contributors to LD–mitochondria interactions. VPS13D likely facilitates lipid transfer between these organelles [59]. MIGA2 binds to membrane proteins VAP-A or VAP-B in the ER and promotes triglyceride synthesis from non-lipid precursors in adipocytes [60]. It connects mitochondrial neo-lipogenic reactions with triacylglycerol production in the ER, enhancing lipid storage in LDs. MIGA2’s widespread presence suggests its importance in maintaining lipid and energy homeostasis across various cell types [16,59].

## 4. Physiological Relevance of LD–Mitochondria Contacts

The interactions between LDs and mitochondria are not just a biochemical curiosity; they play pivotal roles in maintaining physiological homeostasis. Understanding these interactions is crucial for appreciating how cells manage energy resources, respond to oxidative stress, and maintain overall health.

### 4.1. Role in Lipid Transfer and Fatty Acid Oxidation

The interplay between LDs and mitochondria is central to cellular energy homeostasis, particularly in the context of lipid transfer and fatty acid oxidation. This relationship is crucial for understanding metabolic processes, from basic cellular function to the pathogenesis of metabolic diseases.

LDs serve as the primary storage sites for neutral lipids, mainly triglycerides and cholesteryl esters, in the cell. Under conditions of energy surplus, such as excessive caloric intake, LDs accumulate lipids, while in times of energy demand, these lipids are mobilized for use as fuel [61]. The regulation of lipid storage and mobilization within LDs is a tightly controlled process, influenced by hormonal signals and cellular energy needs. Mitochondria are the primary site for fatty acid oxidation, a process critical for converting stored lipids into usable energy [12]. Fatty acid oxidation involves the breakdown of fatty acids into acetyl-CoA, which then enters the tricarboxylic acid cycle, ultimately leading to the production of ATP. This process is especially important during periods of fasting or prolonged exercise, when glucose availability is limited [62].

The first step in lipid mobilization is lipolysis, where triglycerides in LDs are hydrolyzed into free fatty acids and glycerol. Enzymes such as adipose triglyceride lipase (ATGL) and hormone-sensitive lipase (HSL) are key players in this process [63]. Once released, FFAs undergo activation by acyl-CoA synthetase enzymes, converting them into fatty acyl-CoA, which is necessary for their subsequent transport into mitochondria. Fatty acyl-CoA is then transported into the mitochondria by carnitine palmitoyltransferase I located on the outer mitochondrial membrane [64]. Inside the mitochondria, fatty acyl-CoA is converted back into fatty acyl-carnitine, which undergoes β-oxidation [65].

The efficiency of lipid transfer is significantly influenced by the proximity and dynamics of LD–mitochondria contacts. These contacts ensure a streamlined and efficient transfer of fatty acids, minimizing the loss of lipids and maintaining a high rate of fatty acid oxidation [66]. The transfer of lipids and their subsequent oxidation in mitochondria are tightly regulated processes influenced by several factors, including nutritional status, hormonal regulation, and cellular energy sensors. In states of nutrient excess, lipid synthesis and storage are upregulated, leading to a decrease in fatty acid oxidation. Conversely, during nutrient deprivation, lipolysis is enhanced, and fatty acid oxidation is upregulated [67]. Hormones like insulin and glucagon play significant roles. Insulin suppresses lipolysis and promotes lipid storage, while glucagon stimulates lipolysis and increases fatty acid oxidation [68]. AMP-activated protein kinase (AMPK) and other cellular energy sensors detect the energy status of the cell and adjust lipid metabolism accordingly. Activation of AMPK during low-energy states leads to enhanced lipolysis and increased fatty acid oxidation [69].

The LD–mitochondria contacts serve as critical conduits for the transfer of lipids, especially fatty acids, facilitating their oxidation in mitochondria. This transfer is crucial for maintaining cellular energy balance. The proximity of LDs to mitochondria minimizes the diffusion distance, allowing for the efficient transfer of fatty acids [70]. Carrier proteins, possibly including those yet unidentified, are thought to play a role in shuttling these fatty acids across the contact sites. The breakdown of triglycerides into free fatty acids within LDs is the first step in this transfer. Once inside the mitochondria, fatty acids undergo β-oxidation, a process that breaks them down into acetyl-CoA. This acetyl-CoA then enters the TCA cycle, ultimately leading to the production of ATP. The efficiency of fatty acid oxidation is highly dependent on the availability of fatty acids, which is in turn influenced by the dynamics of LD–mitochondria contacts [71]. For energy-intensive processes such as phospholipid biosynthesis and membrane remodeling, substantial amounts of ATP are required. The interactions between LDs and mitochondria, which promote energy production, could support these energy-demanding processes. It is plausible that they have a potential role in facilitating phospholipid biosynthesis and membrane remodeling by ensuring efficient energy supply.

### 4.2. Influence on Cellular Energy Homeostasis

LDs and mitochondria work in concert to balance energy storage and utilization. LDs, as energy storage organelles, accumulate lipids in the form of triglycerides during times of energy surplus. Mitochondria, on the other hand, are the main sites for energy production through fatty acid oxidation. The contacts between LDs and mitochondria facilitate the efficient transfer of fatty acids from LDs to mitochondria, ensuring a rapid response to energy demands [72]. These interactions are central to metabolic flexibility—the ability of a cell to switch between glucose and lipid metabolism depending on availability. The coupling between LDs and mitochondria adjusts in response to nutritional status, exercise, fasting, and other physiological conditions, thereby playing a key role in metabolic health [73]. This hormonal interplay is crucial in conditions such as the postprandial state or during fasting, effectively managing the switch between lipid storage and lipid oxidation [74].

### 4.3. Impact on Oxidative Stress and Redox Signaling

Mitochondria are major sites of ROS production during oxidative phosphorylation. Under normal conditions, ROS serve as signaling molecules, but excessive ROS can lead to oxidative stress and cellular damage [75]. LDs can interact with mitochondria to modulate ROS levels. By supplying fatty acids for β-oxidation, LDs influence the rate of electron transport and subsequent ROS production. Furthermore, LDs can sequester lipid peroxidation products, reducing the burden of oxidative stress on cells [76]. The interplay between LDs and mitochondria is crucial in redox signaling. This cross-talk affects various cellular processes, including the activation of transcription factors like NRF2, which regulates the expression of antioxidant genes, thereby playing a role in the cellular antioxidant response [71].

The ability of cells to adapt to metabolic stress is heavily reliant on the functional cooperation between LDs and mitochondria. This includes the response to nutrient deprivation, physical activity, and changes in metabolic demands, where the efficient use of stored lipids becomes critical for cell survival [38]. Emerging research suggests that LD–mitochondria interactions may have implications in aging and longevity. Efficient energy utilization and the balance in redox homeostasis contribute to cellular longevity, while dysregulation in these processes is associated with age-related diseases [77]. Understanding LD–mitochondria contacts open avenues for therapeutic interventions in metabolic diseases. Targeting these interactions could offer strategies to enhance metabolic flexibility, manage oxidative stress, and improve cellular health in conditions like obesity, diabetes, and cardiovascular diseases [78,79].

### 4.4. Role in Promoting Mitophagy

Mitophagy is the process by which defective mitochondria are eliminated via lysosomes, and elevated levels of mitochondrial autophagy indicate altered metabolism [80]. Researchers investigated metabolic adaptations in cells treated with deferiprone (DFP), a therapeutic iron chelator known to induce PINK1-PRKN-dependent mitophagy [81]. They found that iron depletion remodels lipid metabolism within minutes. DGAT1-dependent LD biosynthesis occurs upstream of mitochondrial turnover, and many LDs come into contact with mitochondria after iron chelation. Inhibition of DGAT1 limits mitophagy through lysosomal dysfunction. In vivo deficiency of mdy/DGAT1 impairs mitochondrial autophagy and motility in *Drosophila* neuronal cells [19]. Long et al. also discovered that mitophagy requires LD expansion mediated by DGAT1 [82]. The researchers measured different stages of the mitochondrial autophagy process in DFP-treated cells in the presence of a DGAT1 inhibitor and in Drosophila lacking the DGAT1 homologue. They found that DGAT1 inhibition reduces mitochondrial autophagy by disrupting lysosomal homeostasis rather than impairing the recruitment of mitochondria into the autophagosome. Mitophagy is diminished because DGAT1 inhibition leads to an increase in non-esterified fatty acid (NEFA), altering the shape and distribution of lysosomes [82]. These changes may impede the fusion of lysosomes with mitochondrial autophagosomes, thereby hindering the clearance of mitochondria by mitochondrial autophagy. Furthermore, mitochondria attached to the few remaining LDs after DGAT1 inhibition are highly susceptible to NEFA-induced damage because their ability to oxidize NEFA is reduced.

## 5. Human Diseases Associated with Alterations in LD–Mitochondria Interaction

**Figure 3:**
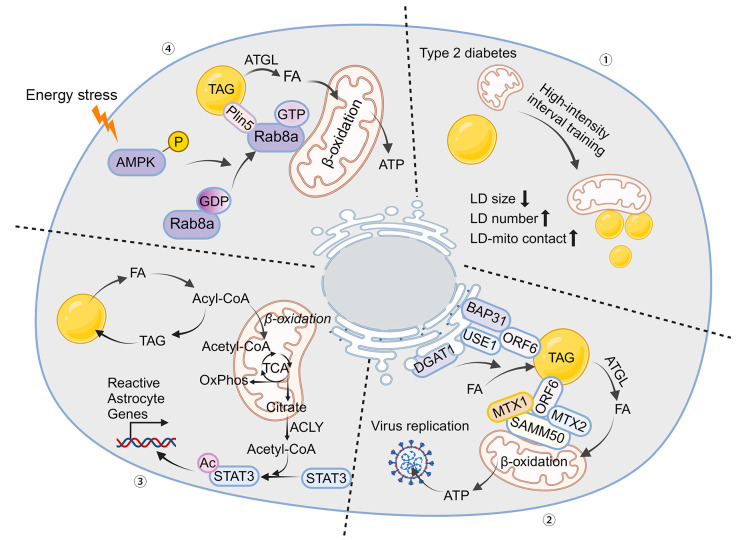
Role of LD–Mitochondria Interaction in Diverse Diseases. (1) Type 2 diabetes: Excessive storage of lipid droplets (LDs) in skeletal muscle is a hallmark of type 2 diabetes. High-intensity interval training (HIIT) exercise alters the size, subcellular distribution, and mitochondrial content of LDs, improving the deficiency of intramuscular LDs. (2) Viral replication: The ORF6 protein inserts into the LD lipid monolayer through its two amphipathic helices. It interacts with endoplasmic reticulum (ER) membrane proteins BAP31 and USE1 to mediate the formation of ER–LD contacts. Additionally, ORF6 connects mitochondria to LDs by interacting with the SAM complex in the mitochondrial outer membrane, promoting cellular lipolysis and LD biogenesis, reprogramming lipid fluxes, and facilitating viral replication. (3) Astrocyte reactivity: When fatty acid load exceeds the oxidative phosphorylation (OxPhos) capacity of astrocytes, elevated acetyl-CoA levels induce astrocyte reactivity by enhancing STAT3 acetylation and activation. (4) Fatty acid utilization in skeletal muscle: In rat skeletal muscle cells, the energy sensor AMPK increases the GTP-binding activity of Rab8a, facilitating LD–mitochondria interactions by binding to PLIN5 under starvation conditions. The assembly of the Rab8a-PLIN5 tethering complex recruits ATGL, mobilizing and transferring long-chain fatty acids (LCFAs) from LDs to mitochondria for β-oxidation. Rab8a deficiency in a mouse model impairs fatty acid utilization and reduces exercise endurance. The arrows mean decrease or increase.

The physiological interplay between LDs and mitochondria, essential for maintaining cellular homeostasis, can have profound pathological implications when dysregulated. This section delves deep into the molecular underpinnings of these interactions in various diseases, highlighting their critical role in pathology (Figure 3).

### 5.1. Obesity and Type 2 Diabetes

Obesity is characterized by excessive accumulation of lipids in adipocytes, leading to hypertrophic LDs and altered mitochondrial function. This imbalance results in a decreased efficiency of mitochondrial fatty acid oxidation, contributing to a surplus of circulating free fatty acids and their deposition in non-adipose tissues, a condition known as lipotoxicity [83]. In skeletal muscle and the liver, this ectopic fat accumulation causes insulin resistance by interfering with insulin signaling pathways, mainly through the activation of inflammatory pathways and the production of cytokines like TNF-α. Furthermore, the oversupply of FFAs to the liver exacerbates insulin resistance and promotes gluconeogenesis, aggravating hyperglycemia [84].

In type 2 diabetes, dysregulation of LD–mitochondria interactions significantly contribute to insulin resistance and β-cell dysfunction. In peripheral tissues, impaired mitochondrial oxidative capacity leads to incomplete fatty acid oxidation, causing harmful lipid intermediates such as diacylglycerols and ceramides to accumulate, which inhibits insulin signaling. In pancreatic β-cells, mitochondrial dysfunction impacts ATP production, which is crucial for insulin secretion. Faulty LD dynamics result in lipid accumulation in β-cells, further impairing insulin secretion and contributing to hyperglycemia [45,85,86]. Excessive LD storage in skeletal muscle is a hallmark of type 2 diabetes [87]. However, LD morphology shows substantial subcellular heterogeneity and varies among individual muscle fibers. A comprehensive single-fiber morphology analysis using quantitative transmission electron microscopy compared type 2 diabetic patients to non-diabetic obese and lean controls. Researchers found that excessive lipid storage in the muscles of type 2 diabetic patients was due to oversized LDs in different muscle fibers and a lack of LDs in specific locations relative to mitochondria [86]. High-intensity interval training altered the size, subcellular distribution, and mitochondrial content of LDs, improving the intramuscular LD deficit. Additionally, the physical contact between LDs and mitochondrial membranes indicates dysregulated organelle interactions in the diabetic state [88]. Type 2 diabetes is considered a metabolic disease characterized by significant cellular heterogeneity in intramuscular lipid storage, emphasizing the importance of single-cell techniques in clinical research.

### 5.2. Hepatic Lipotoxicity

Aflatoxin B1 (AFB1) is one of the most toxic mycotoxins commonly found in food contaminants, primarily targeting the liver [89]. It poses a significant threat to global food safety and public health. Researchers examined the potential hepatic lipotoxicity resulting from AFB1 exposure using both in vitro and in vivo models to evaluate the public health risks associated with high dietary AFB1 intake [90]. The study showed that low doses of AFB1 (1.25 μM for 48 h, about one-fifth of the IC50 for HepG2 and HepaRG cells, which were 5.995 μM and 5.266 μM, respectively) significantly induced hepatic steatotoxicity. This was characterized by abnormal growth of LDs, increased mitochondrial–LD contact, disrupted phagocytosis, and lipid accumulation. AFB1 exposure enhanced mitochondrial–LD contact through the interaction of mitochondrial p53 (mito-p53) and the LD-associated protein perilipin 2 (PLIN2), leading to lipid accumulation in hepatocytes. Targeted inhibition of mito-p53, knockdown of PLIN2, and the application of rapamycin effectively promoted lysosome-dependent lipophagy and reduced hepatic lipotoxicity and liver injury caused by AFB1 exposure [90]. In summary, interactions between mito-p53 and PLIN2 regulate lipid homeostasis in AFB1-induced hepatotoxicity through a network involving mitochondria, LDs, and lysosomes. This unique trio of organelles works in concert, offering new insights for targeted interventions in inter-organellar lipid sensing and trafficking to mitigate hepatic lipotoxicity induced by harmful substances.

In NAFLD, the disrupted balance between lipid acquisition, storage, and oxidation in hepatocytes leads to hepatic steatosis. The main contributors are an increased influx of free fatty acids (FFAs) from adipose tissue and de novo lipogenesis in the liver, worsened by decreased fatty acid oxidation due to mitochondrial dysfunction. This persistent FFA overload induces mitochondrial stress, oxidative damage, and eventually apoptosis, facilitating the progression from simple steatosis to non-alcoholic steatohepatitis (NASH), characterized by inflammation and fibrosis. A hallmark of NAFLD is the excessive accumulation of LDs [91]. Interactions between LDs and mitochondria are crucial for lipid metabolic homeostasis. Aerobic exercise reduces the size of LDs bound to mitochondria and decreases the number of LD–mitochondrial contacts. There is a positive correlation between the number of LD–mitochondrial contacts and the severity of NAFLD as well as the size of mitochondria-bound LDs. Cellular fractionation studies have shown enhanced ATP-coupled respiration and fatty acid oxidation in the periportal mitochondria of the livers of exercising mice on a high-fat diet compared to their sedentary counterparts, despite similar body weights. Exercise increases fatty acid oxidation and mitofusin-2 abundance in mitochondria through mechanisms involving mitochondrial membrane curvature and the abundance of saturated lipids. Consequently, the ablation of hepatic mitofusin-2 prevents the exercise-induced enhancement of fatty acid oxidation in periportal mitochondria [92].

### 5.3. Skeletal Muscle Exercise Tolerance

The dynamic interactions between lipid droplets (LDs) and mitochondria are essential for mobilizing long-chain fatty acids (LCFAs) from LDs to mitochondrial β-oxidation in skeletal muscle during energy stress. Rab8a has been identified as a mitochondrial LD receptor that forms a tethered complex with the LD-associated protein PLIN5 in skeletal muscle [1]. In rat L6 skeletal muscle cells, the energy sensor AMPK enhances the GTP-binding activity of Rab8a, facilitating LD–mitochondrial interactions by binding to PLIN5 under starvation conditions. The assembly of the Rab8a-PLIN5 tethering complex also recruits ATGL, which mobilizes and transfers LCFAs from LDs to mitochondria for β-oxidation. In a mouse model, Rab8a deficiency impaired fatty acid utilization and reduced exercise endurance [1]. These findings may help elucidate the regulatory mechanisms underlying the beneficial effects of exercise on lipid homeostasis.

### 5.4. Cardiovascular Diseases (CVDs)

In the context of cardiovascular disease (CVD), dysfunctional interactions between LDs and mitochondria have significant implications for atherogenesis and heart failure. In atherosclerosis, the accumulation of oxidized low-density lipoprotein (LDL) particles within macrophages leads to foam cell formation, a critical event in plaque development. The ability of these cells to manage lipid overload via LDs and mitochondria is crucial in preventing plaque formation. In the myocardium, imbalances in LD dynamics and mitochondrial oxidative capacity result in excessive lipid accumulation, leading to lipotoxic cardiomyopathy [93]. Vascular smooth muscle cell (VSMC) senescence accelerates atherosclerosis progression through lipid-mediated mitochondrial dysfunction and oxidative stress. Research has shown that SRT1720 improves mitochondrial DNA (mtDNA) damage and enhances mitochondrial repair in VSMCs affected by oleic acid (OA)-induced mitochondrial dysfunction. Additionally, SRT1720 reduces mitochondrial reactive oxygen species levels in OA-treated VSMCs. SRT1720 significantly elevates the expression levels of Sirtuin 1 (SIRT1) and peroxisome proliferator-activated receptor γ coactivator 1α (PGC-1α). However, pre-treatment with EX527 and SR-18292 prior to SRT1720 administration did not reverse senescence, the inflammatory response, or the atherosclerotic phenotype in VSMCs [94]. The upregulation of SIRT1 and deacetylation of PGC-1α by SRT1720 restores mitochondrial function, inhibiting VSMC senescence and the expression of atherosclerosis-related proteins and phenotypes. Through SIRT1-mediated deacetylation of the PGC-1α pathway, SRT1720 mitigates OA-induced atherosclerosis associated with VSMC senescence and mitochondrial dysfunction [94,95].

### 5.5. Virus Replication

LDs form inter-organelle contacts with the endoplasmic reticulum (ER) to facilitate their biogenesis, while contacts between LDs and mitochondria enhance the β-oxidation of fatty acids contained within them [96]. Viruses have been shown to utilize LDs to promote viral production [97]. Researchers discovered that the coronavirus ORF6 protein targets LDs, localizes at mitochondria–LD and ER–LD contact sites, and regulates both LD biogenesis and lipolysis [37]. At the molecular level, ORF6 inserts into the LD lipid monolayer via its two amphipathic helices. Additionally, ORF6 interacts with ER membrane proteins B cell receptor associated protein 31 (BAP31) and unconventional SNARE in the ER 1 (USE1) to mediate the formation of ER–LD contacts. ORF6 also interacts with the SAM complex in the mitochondrial outer membrane to link mitochondria to LDs. Through these interactions, ORF6 promotes cellular lipolysis and LD biogenesis, thereby reprogramming lipid fluxes in the host cell and enhancing virulence [37].

### 5.6. Neurodegenerative Diseases

Astrocytes play a crucial role in supporting neurons, and their phenotypic shifts are associated with the onset of neurodegenerative diseases [98]. Metabolically, astrocytes exhibit low mitochondrial oxidative phosphorylation (OxPhos) activity. Research has shown that the brain relies significantly on astrocyte OxPhos for fatty acid degradation and maintaining lipid homeostasis. Dysregulation in astrocyte OxPhos leads to lipid droplet (LD) accumulation, triggering neurodegenerative changes characteristic of Alzheimer’s disease, such as synaptic loss, neuroinflammation, demyelination, and cognitive deficits [99]. When the fatty acid load exceeds the OxPhos capacity of astrocytes, elevated acetyl-CoA levels induce astrocyte reactivity by promoting STAT3 acetylation and activation [17].

Lipid-rich reactive astrocytes enhance fatty acid oxidation and oxidative stress in neurons, activate microglia through IL-3 signaling, and suppress the biosynthesis of fatty acids and phospholipids essential for myelin repair. The progressive mitochondrial dysfunction in astrocytes sequentially triggers neuroinflammation and neurodegeneration. The link between metabolic dysregulation and Alzheimer’s disease (AD) pathology is becoming increasingly clear. In AD, neurons show altered lipid metabolism and mitochondrial dysfunction, with dysfunctional LDs leading to toxic lipid accumulation, and mitochondrial impairment resulting in reduced ATP production and increased oxidative stress [100]. These factors contribute to amyloid-β accumulation and tau pathology. LD–mitochondria interactions in neurons are crucial for managing lipid homeostasis and mitochondrial health, impacting AD progression [101].

Parkinson’s disease (PD) is characterized by the degeneration of dopaminergic neurons in the substantia nigra. A well-established feature in PD is mitochondrial dysfunction, specifically impaired complex I activity of the electron transport chain. Additionally, changes in lipid metabolism, including LD dynamics, have been observed in PD models. These alterations in LD–mitochondria interactions may contribute to neuronal death by disrupting energy homeostasis and increasing susceptibility to oxidative stress [102,103].

## 6. Therapeutic Perspectives

The intricate relationship between LDs and mitochondria presents a novel avenue for therapeutic intervention, particularly in metabolic, cardiovascular, and neurodegenerative diseases. This section explores the potential of targeting these interactions, recent advancements in the field, and the challenges faced in translating these findings into clinical practice.

The dysregulation of LD–mitochondria interactions play a central role in the pathophysiology of various diseases, making it a compelling target for therapeutic intervention. Modulating these interactions could restore metabolic balance, reduce oxidative stress, and improve cellular function [104]. Approaches to modulate these interactions include enhancing mitochondrial fatty acid oxidation, regulating lipid mobilization from LDs, and restoring the structural integrity of mitochondria [49]. Pharmacological agents that target key enzymes or regulatory proteins involved in LD dynamics or mitochondrial function are under investigation [105]. In metabolic diseases like obesity and type 2 diabetes, therapies aimed at enhancing lipid oxidation in mitochondria or reducing lipid accumulation in LDs could improve insulin sensitivity and glucose homeostasis. For instance, activating AMPK has been shown to stimulate mitochondrial biogenesis and fatty acid oxidation [106].

Recent research has identified several potential drug targets within the LD–mitochondria axis. These include enzymes involved in lipid metabolism (e.g., lipases), proteins regulating LD–mitochondria contacts (e.g., perilipins), and mitochondrial proteins involved in fatty acid transport and oxidation (e.g., CPT1) [47,107,108]. The development of small molecules that can specifically modulate the activity of these targets is underway. Such molecules have the potential to fine tune the metabolic processes mediated by LD–mitochondria interactions. AMPK activators, such as metformin, have shown promise in enhancing mitochondrial function and energy expenditure. They are being explored for their broader implications in metabolic diseases, extending beyond glycemic control [40].

Despite the promise, translating these findings from bench to bedside poses significant challenges. These include the complexity of LD–mitochondria interactions, the need for tissue-specific targeting, and potential off-target effects [109,110]. Future research is directed towards understanding the precise molecular mechanisms governing LD–mitochondria interactions in different disease contexts. This knowledge is crucial for designing more effective and targeted therapeutic strategies. Considering the variability in metabolic responses among individuals, a personalized medicine approach, taking into account genetic and epigenetic factors influencing LD–mitochondria dynamics, could be pivotal in the effective management of metabolic and degenerative diseases [111].

## 7. Conclusions and Perspectives

The exploration of LD and mitochondria interactions has unveiled a fascinating dimension of cellular biology, emphasizing its crucial role in energy homeostasis, metabolic regulation, and disease pathogenesis. We have journeyed through the dynamic world of these organelle interactions, uncovering their pivotal role in balancing energy storage and utilization, adapting to metabolic stress, and maintaining cellular health. This journey has also revealed the profound implications of these interactions in a range of diseases, including metabolic disorders, liver and cardiovascular diseases, and neurodegenerative conditions. The intricacies of LD–mitochondria contacts, once a niche area of cellular biology, have emerged as a potential goldmine for therapeutic interventions, offering novel strategies for tackling metabolic and degenerative diseases.

Despite significant advancements, critical gaps remain in our understanding. The precise molecular mechanisms governing LD–mitochondria interactions are yet to be fully elucidated. Future research should aim to uncover these mechanisms, exploring the potential of integrative systems biology approaches to provide a holistic understanding. Additionally, the tissue-specific dynamics of these interactions and their implications in organ-specific diseases present a fertile ground for further investigation. Translating these basic research findings into clinical applications is a paramount next step, necessitating a concerted effort to develop targeted therapeutics and evaluate them in clinical trials. The adoption of personalized medicine approaches, considering individual genetic and metabolic profiles, will be key in optimizing these treatment strategies.

As we stand on the cusp of new discoveries, the study of LD–mitochondria interactions beckon an interdisciplinary approach. Integrating insights from cell biology, biochemistry, pharmacology, and clinical science is imperative to fully harness the potential of this field. The future promises not just a deeper understanding of cellular metabolism but also novel, effective therapeutic approaches for a range of challenging diseases. The journey of exploring LD–mitochondria interactions, therefore, is not just a path to academic enrichment but a beacon of hope for advancements in human health and medicine.

This review has traversed the complex and dynamic landscape of LD and mitochondria interactions, shedding light on their pivotal role in cellular metabolism and highlighting their significance in health and disease. We have delved into the mechanisms of these organelle interactions, their physiological relevance, and the profound impact their dysregulation has on a spectrum of diseases, including metabolic disorders, liver and cardiovascular diseases, and neurodegenerative conditions. Furthermore, this exploration has unveiled promising therapeutic opportunities, emphasizing the potential of targeting LD–mitochondria interactions as a novel strategy for disease intervention. As we conclude, it is evident that understanding the intricate dance between LDs and mitochondria is not just crucial for deciphering cellular metabolic processes but also holds the key to unlocking new avenues in disease treatment and prevention, heralding a new era in medical research and therapeutic development.
